# Ventricular Arrhythmia and Cardiac Fibrosis in Endurance Experienced Athletes (VENTOUX)

**DOI:** 10.1161/CIRCIMAGING.125.018470

**Published:** 2025-07-17

**Authors:** Wasim Javed, Ioannis Botis, Ze Min Goh, Mubien Shabi, Benjamin Brown, Raluca Tomoaia, Maryum Farooq, Eylem Levelt, Lee Graham, John Gierula, Peter Kellman, John P. Greenwood, Sven Plein, Peter P. Swoboda

**Affiliations:** 1 Leeds Institute of Cardiovascular and Metabolic Medicine, University of Leeds/Leeds Teaching Hospitals National Health Service Trust, United Kingdom (W.J., I.B., Z.M.G., M.S., B.B., R.T., M.F., L.G., J.G., J.P.G., S.P., P.P.S.).; 2 Iuliu Hațieganu University of Medicine and Pharmacy, Cluj-Napoca, Romania (R.T.).; 3 Baker Heart and Diabetes Institute and University of Melbourne, Australia (E.L., J.P.G.).; 4 National Institutes for Health, National Heart, Lung, and Blood Institute, Bethesda, MD (P.K.).

**Keywords:** athletes, dilatation, fibrosis, perfusion

## Abstract

**BACKGROUND:**

Sudden cardiac death due to primary arrhythmia is a leading cause of mortality in athletes, predominantly affecting older male athletes. Myocardial fibrosis is strongly associated with arrhythmogenesis in nonischemic cardiomyopathy, but its clinical significance in asymptomatic endurance athletes is unknown. We aimed to investigate whether myocardial fibrosis on cardiovascular magnetic resonance in asymptomatic veteran male athletes was associated with incident ventricular arrhythmia on long-term implantable loop recorder.

**METHODS:**

Prospective observational cohort study involving 106 asymptomatic male competitive cyclists/triathletes (aged ≥50 years) who undertook ≥10 h/wk of exercise for ≥15 years. Exclusion criteria were any preexisting cardiovascular disease. Participants underwent clinical assessment, stress-perfusion late gadolinium enhancement-cardiovascular magnetic resonance, exercise testing, and implantable loop recorder implantation to detect ventricular arrhythmia. Athletes were followed up for the primary end point of incident ventricular arrhythmia.

**RESULTS:**

A total of 50/106 (47.2%) athletes had focal myocardial fibrosis (all nonischemic distribution) on cardiovascular magnetic resonance predominantly affecting the basal inferolateral left ventricular segment. During follow-up (median 720 days), 23/106 (21.7%) athletes experienced ≥1 ventricular arrhythmic episode; 3/106 (2.8%) sustained ventricular tachycardia, and 20/106 (18.9%) nonsustained ventricular tachycardia. Myocardial fibrosis (hazard ratio, 4.7 [95% CI, 1.8–12.8]; *P*=0.002) and greater left ventricular end-diastolic volume indexed (hazard ratio, 1.4 [95% CI, 1.1–1.9]; *P*=0.02) were associated with an increased risk of incident ventricular arrhythmia, but right ventricular insertion point late gadolinium enhancement was not (hazard ratio, 1.7 [95% CI 0.6–5.1]; *P*=0.32). Myocardial fibrosis remained predictive after adjusting for left ventricular end-diastolic volume indexed (hazard ratio, 4.7 [95% CI, 1.7–12.7]; *P*=0.002).

**CONCLUSIONS:**

In male veteran endurance athletes, myocardial fibrosis was independently associated with the onset of ventricular arrhythmia, even after adjusting for left ventricular dilatation. Right ventricular insertion point late gadolinium enhancement was not associated with ventricular arrhythmia. Further studies are needed to establish whether myocardial fibrosis itself is arrhythmogenic or a marker of an underlying cardiomyopathic process.

**CLINICAL PERSPECTIVE**

In this prospective study, myocardial fibrosis on cardiovascular magnetic resonance imaging was independently associated with the risk of ventricular arrhythmia in healthy, asymptomatic veteran male endurance athletes. Other predictors of ventricular arrhythmia included left ventricular dilatation, and exercise-induced premature ventricular contractions. As the incidence of ventricular arrhythmia may be associated with sudden cardiac arrest, the presence of myocardial fibrosis, left ventricular dilatation and exercise-induced premature ventricular contractions may play a role in indirectly predicting the risk of sudden cardiac arrest among certain athletes. However, further studies are needed to confirm this and to determine whether athletes with myocardial fibrosis on cardiovascular magnetic resonance have a concealed form of cardiomyopathy.

**See Editorial by McMenamin and Ruberg**

Physical exercise is unequivocally beneficial for cardiovascular health. However, sudden cardiac death (SCD) is a leading cause of mortality in athletes and is believed to predominantly affect older male recreational athletes.^1^ Currently, the underlying mechanisms of SCD in athletes are unclear, and an improved understanding is needed to enhance risk stratification and guide safe participation in sport. This need is especially true for older athletes, who not only have the highest risk but also constitute the age category that undertakes the most recreational exercise.^2^

Postmortem studies of athletes who have suffered SCD indicate left ventricular (LV) fibrosis as one of the strongest predictors of SCD.^3^ These athletes typically exhibit physiological cardiac remodeling but no overt pathological features, and hence, they are often undetected by conventional screening strategies.

Cardiovascular magnetic resonance (CMR) imaging can detect and quantify LV fibrosis in vivo with high accuracy. A significant proportion of lifelong endurance athletes, in particular older male athletes, exhibit myocardial fibrosis on CMR, which commonly affects the basal inferolateral myocardial segment in a nonischemic distribution.^4,5^ Similar focal fibrosis is known to be arrhythmogenic and associated with adversity in nonischemic cardiomyopathy, but its clinical relevance in asymptomatic athletes is unknown.^6,7^ While retrospective studies imply a correlation between nonischemic basal inferolateral LV fibrosis and arrhythmogenesis in competitive athletes who present with ventricular arrhythmia, there is a paucity of prospective data linking nonischemic fibrosis with ventricular arrhythmia in healthy, asymptomatic athletes.^8,9^

We aimed to determine whether focal myocardial fibrosis in asymptomatic veteran endurance athletes was prospectively associated with the incidence of ventricular arrhythmia on long-term implantable loop recorder (ILR) monitoring.

## Methods

### Study Design

This single-center prospective cohort study was granted ethical approval by the South Yorkshire & Humber NHS Research Ethics Committee and Health Research Authority (21/YH/0231). Participants provided written informed consent and underwent investigations between January 2022 and September 2022 at the University of Leeds, Advanced Imaging Center, Leeds General Infirmary, Leeds, United Kingdom. The data that support the findings of this study are available from the corresponding author upon reasonable request.

### Participant Recruitment

Participants were recruited from sporting clubs/organizations within the United Kingdom via email invitation to their respective club/organization.

#### Inclusion Criteria

Male cyclists/triathletes aged ≥50 years old undertaking ≥10 hours of exercise per week for ≥15 years and regularly competing at local, national, or international level.

#### Exclusion CVD Criteria

Preexisting coronary artery disease (including CVD, tachyarrhythmia, and arterial hypertension), significant medical condition (mild hyperlipidemia defined as requiring the use of a single lipid-lowering medication and without a known inherited lipid disorder, and mild asthma were not considered significant), or contraindication to CMR. Athletes were excluded if they had any cardiac symptoms suggestive of underlying CVD (anginal chest pain, palpitations, exertional dyspnea, and syncope). One hundred and thirty-nine potential participants were screened, of whom 106 were recruited to the study. Reasons for exclusion consisted of not meeting age requirements (n=6) and prior CVD (n=27).

A comparative group of age-matched male nonathletic controls were also recruited to undergo an identical CMR protocol to identify whether the observed prevalence of myocardial fibrosis was specific to athletes. Inclusion criteria: aged ≥50 years (identical to the athlete cohort) and participation in formal exercise of <3 h/wk. Exclusion criteria: known CVD, symptoms suggestive of CVD, and any regular medications.

### Baseline Assessment

Participants underwent physical examination, which included measurement of resting blood pressure and heart rate (HR). A self-reported full medical, sporting, and lifestyle history was documented, and cardiovascular risk factors were recorded, including any prior history of hyperlipidemia and family history of CVD including cardiomyopathy and SCD. Social histories were taken, consisting of smoking status, alcohol and caffeine intake along with any use of performance-enhancing drugs.

All participants underwent blood sampling for full blood count, renal profile, lipid profile, and hemoglobin A1c, and resting 12-lead ECG (MAC500; GE Medical Systems, Milwaukee, WI).

### CMR Protocol

Participants underwent CMR on a 3.0T Magnetom Prisma Siemens system. The CMR scan protocol (Supplemental Material) consisted of:

- Cine imaging in short-axis and multiple long-axis planes for volumetric analysis.
- Adenosine stress and rest quantitative myocardial perfusion to identify myocardial ischemia.
- Pre-and postcontrast T1 mapping to allow estimation of the myocardial extracellular volume fraction.
- T2 mapping to identify inflammation and edema.
- Motion-corrected bright and dark blood late gadolinium enhancement (LGE) in short-axis and multiple long-axis planes to identify and quantify LV fibrosis.

### CMR Analysis

All CMR studies were analyzed using commercially available software (CVI42; Circle Cardiovascular Imaging, Inc, Calgary, Canada; Supplemental Material).

### Implantable Loop Recorder

Participants received a Biomonitor IIIm (Biotronik GmbH & Co, Berlin, Germany) ILR. ILRs were programmed to record tachyarrhythmia faster than the participant’s maximum HR defined on exercise testing for ≥8 consecutive beats. Automated nightly remote downloads were performed by a CardioMessenger Smart mobile unit (Biotronik GmbH & Co). ILRs were left in place for the entirety of the study unless there was a clinical indication or participant request for removal.

### Functional Threshold Power Ramp Exercise Test

With an ILR in place, participants underwent a supervised exercise cycling test on a stationary exercise bicycle (Wattbike Pro, Wattbike, United Kingdom) with ECG monitoring after abstaining from caffeine and strenuous exercise for 24 hours prior (Table S1).

### Follow-Up

Each participant was asked to continue with their normal daily activities and sporting habits. Participants recorded any symptoms via the Biotronik Patient Application (Biotronik GmbH & Co) which was downloaded on participant’s smartphones at the time of ILR implantation.

Participants were followed up with daily monitoring of ILR heart rhythm data for the main outcome of ventricular arrhythmia: ventricular tachycardia (VT) defined as ≥30 seconds of consecutive ventricular beats at >100 bpm and nonsustained VT defined as ≥3 consecutive ventricular beats lasting ≤30 seconds, as per consensus guidelines.^1^ Arrhythmic events were confirmed by a Consultant Electrophysiologist blinded to CMR findings. Any participant who developed a potentially harmful arrhythmia was contacted urgently to assess symptoms and advise them to seek independent medical attention if appropriate. Participants were sent 6-monthly questionnaires to complete, which detailed any new medical diagnoses or medication commenced.

### Statistical Analysis

A prespecified statistical analysis plan was produced before study commencement by a statistician (Leeds Clinical Trials Research Unit). Power calculations were performed to determine the study sample size, which was deemed sufficient to enable a univariate Cox regression model to be fitted on time to ventricular arrhythmia, adjusting for the presence of fibrosis with a 5% participant dropout margin. A multivariable model was also prespecified to include 1 covariate per 10 outcome events.

Statistical analyses were undertaken using SPSS Statistics 29 (IBM SPSS, Armonk, New York). Normality of data was assessed using the Shapiro-Wilk test. Continuous data were presented as mean±SD or median (interquartile range), depending on the normality of the data. Categorical data were presented as frequency (%). Continuous variables were compared using unpaired *t* test or Mann-Whitney *U* test, depending on the normality of the data. Categorical variables were compared using chi-squared test or Fisher exact test. Cox proportional hazard regression analysis and log-rank testing were used to compare the probability of developing ventricular arrhythmia in those with and without myocardial fibrosis. Binomial logistic regression was used to compare variables associated with the presence of myocardial fibrosis to identify potential confounders. A 2-sided *P* value of <0.05 was considered statistically significant in all analyses.

## Results

**Figure 1.**
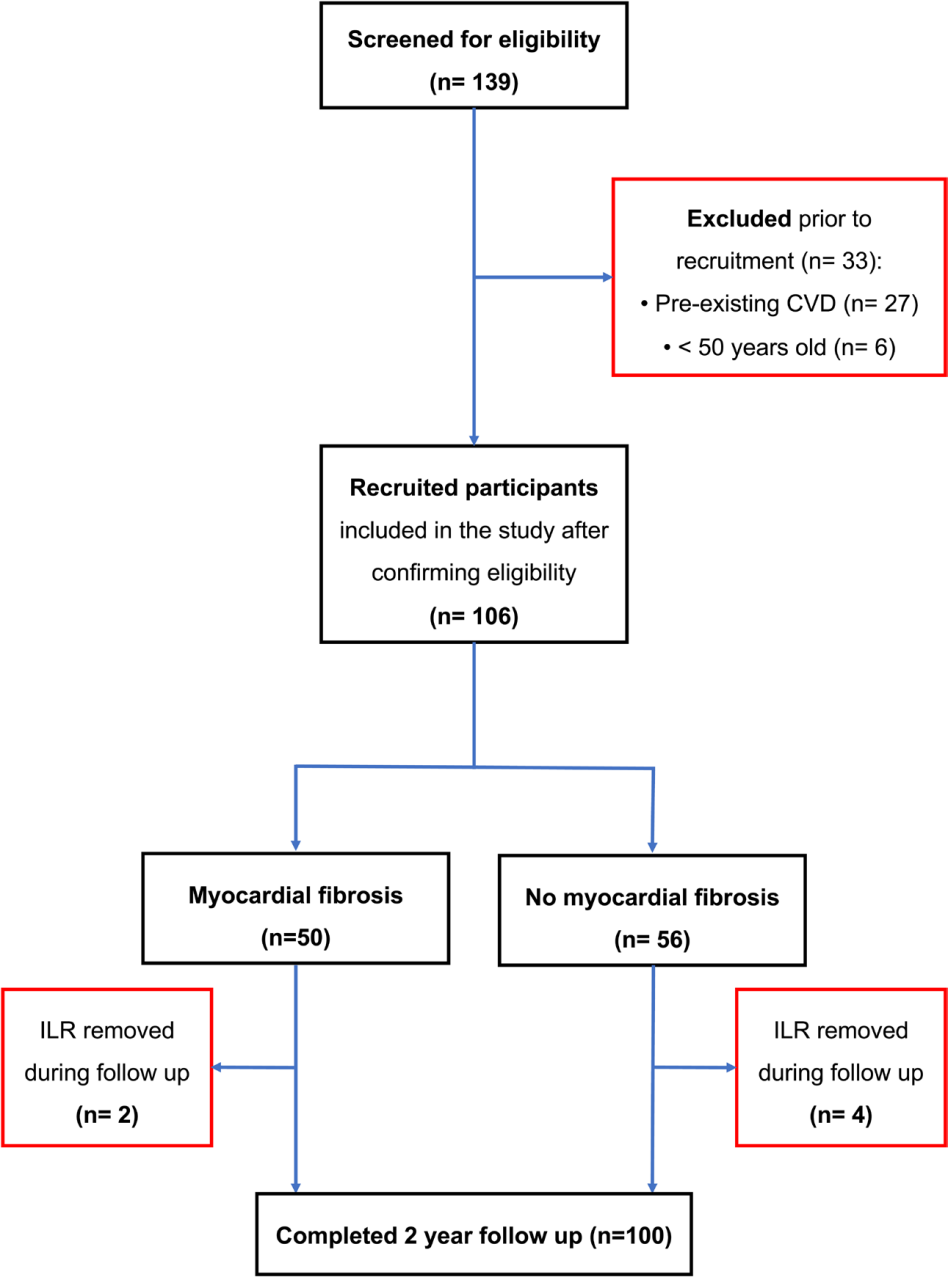
**CONSORT diagram.** Flowchart of participants; 139 athletes were screened, of which 33 were excluded due to prior cardiovascular disease (CVD) or ineligibility based on age. A total of 106 athletes were included, of which 50 had myocardial fibrosis and 56 did not have myocardial fibrosis on cardiovascular magnetic resonance (CMR). During follow-up, 4 athletes underwent implantable loop recorder (ILR) removal in the no fibrosis group and 2 athletes in the fibrosis group.

A total of 106/106 (100.0%) athletes were followed up over a median period of 720 days (interquartile range, 665−756 days). In total, 6/106 (5.7%) requested to have their ILR devices removed during follow-up (median 123 days; Figure 1).

### CMR Characteristic and Prevalence of Myocardial Fibrosis

**Figure 2.**
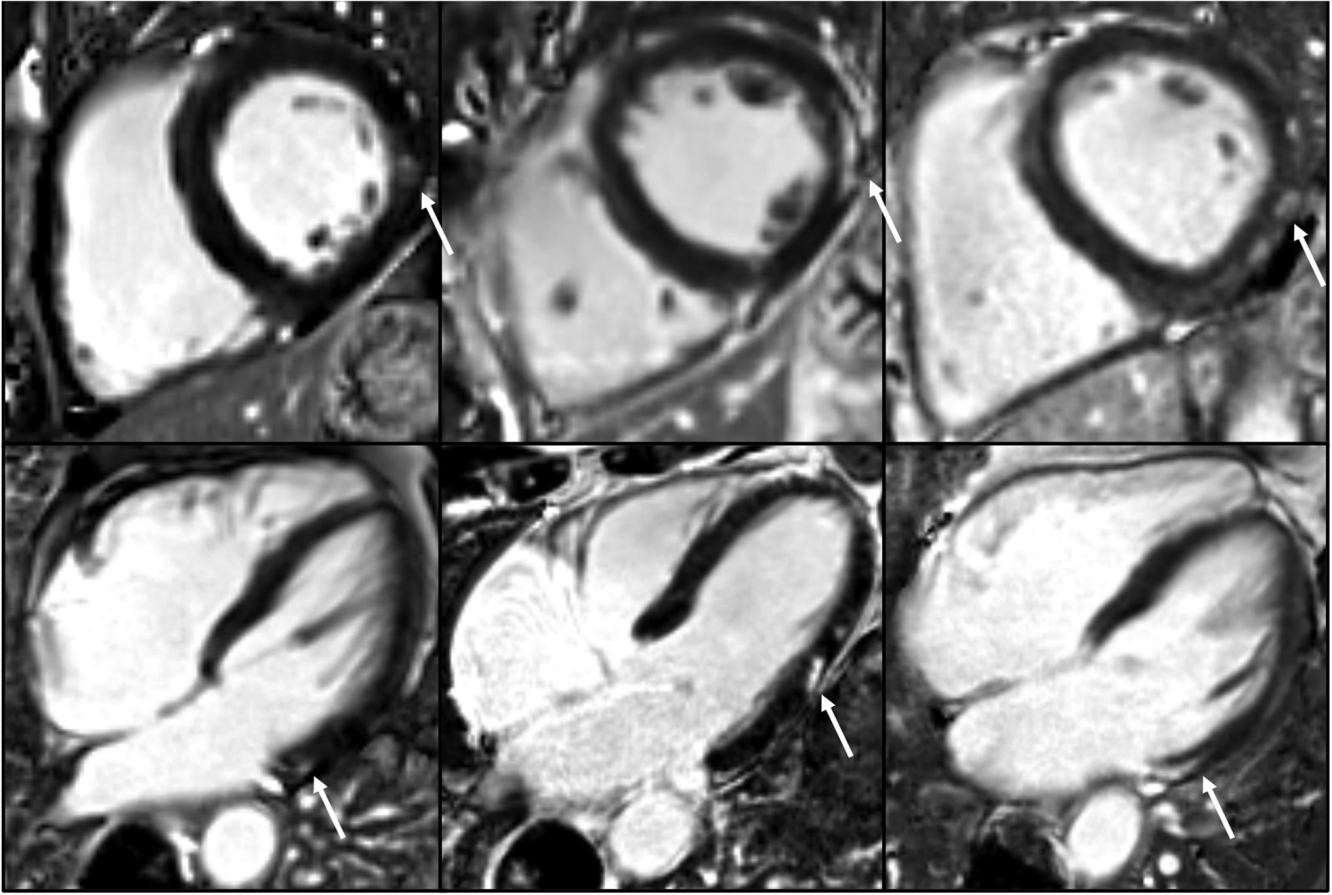
**Athletes with late gadolinium enhancement (LGE).** Bright blood LGE-cardiovascular magnetic resonance (CMR) images of left ventricular (LV) short-axis (SAX; **top**) with corresponding LV long-axis (LAX; **bottom**) images demonstrating fibrosis (white arrow) of the lateral LV segments of a 56-year-old male cyclist (**left**), a 75-year-old triathlete (**middle**), and a 64-year-old cyclist (**right**).

A total of 50/106 (47.2%) athletes had focal myocardial fibrosis on LGE imaging, 50/50 (100%) of which was nonischemic in distribution and predominantly affected the basal inferolateral myocardial segment in 44/50 (88.0%) athletes, with no evidence of myocardial infarction in any athlete (Figure 2). Athletes had a significantly greater prevalence of myocardial fibrosis than nonathletes (50/106 [47.2%] versus 3/27 [11.1%], *P*<0.001; Table S2).

Athletes with fibrosis were slightly older than athletes without fibrosis (61.8±5.8 years versus 57.0±4.5 years, *P*<0.001; Table 1). There were no significant differences in any CMR parameter between those with and without fibrosis including LV end-diastolic volume indexed (LVEDVi) to BSA (110±15 mL/m^2^ versus 106±14 mL/m^2^; *P*=0.23) and LV ejection fraction (55.8±4.1% versus 56.0±4.3%; *P*=0.86), while CMR findings of balanced biventricular dilatation were in keeping with athletic remodeling among both groups.

**Table 1.**
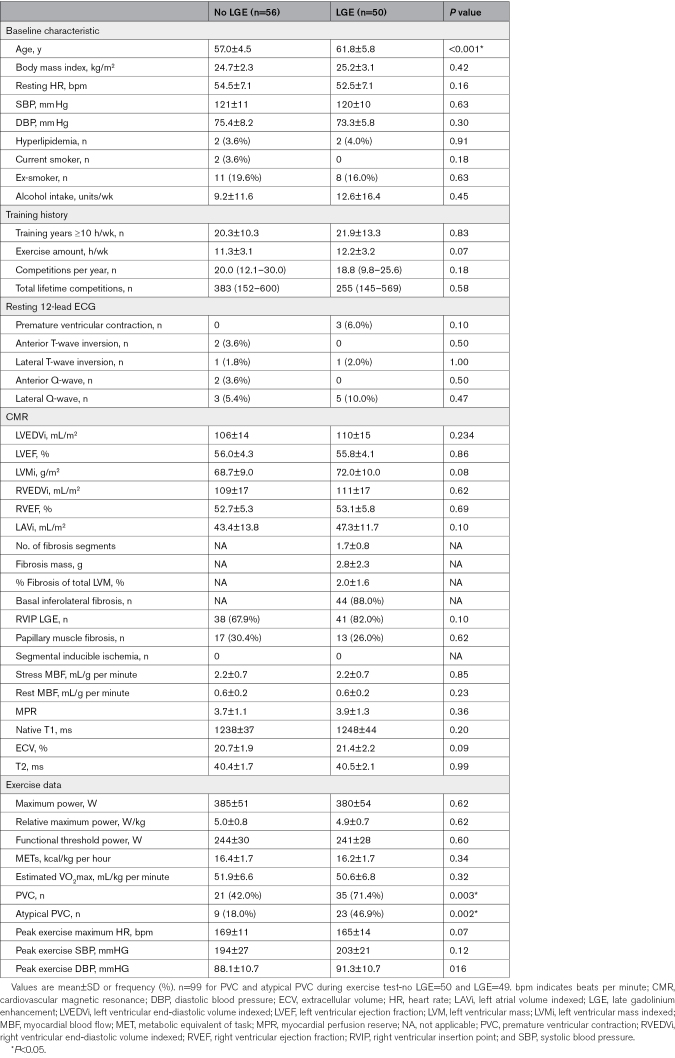
Comparison of Athletes With and Without Myocardial Fibrosis

Indices of quantitative stress perfusion did not differ between athletes with and without myocardial fibrosis, and there was no evidence of inducible perfusion defects in any athlete (stress myocardial blood flow 2.2±0.7 mL/g per minute versus 2.2±0.7 mL/g per minute, *P*=0.85; rest myocardial blood flow 0.6 mL/g per minute ±0.2 versus 0.6 mL/g per minute ±0.2, *P*=0.23; myocardial perfusion reserve 3.9±1.3 versus 3.7±1.1, *P*=0.36).

Athletes with fibrosis exhibited a greater prevalence of premature ventricular contractions (PVCs) during exercise testing than athletes without fibrosis (35/49 [71.4%] versus 21/50 [42.0%]; *P*=0.003) with a greater burden of atypical features (23/49 [46.9%] versus 9/50 [18.0%]; *P*=0.002).

### Incidence of Ventricular Arrhythmia

**Figure 3.**
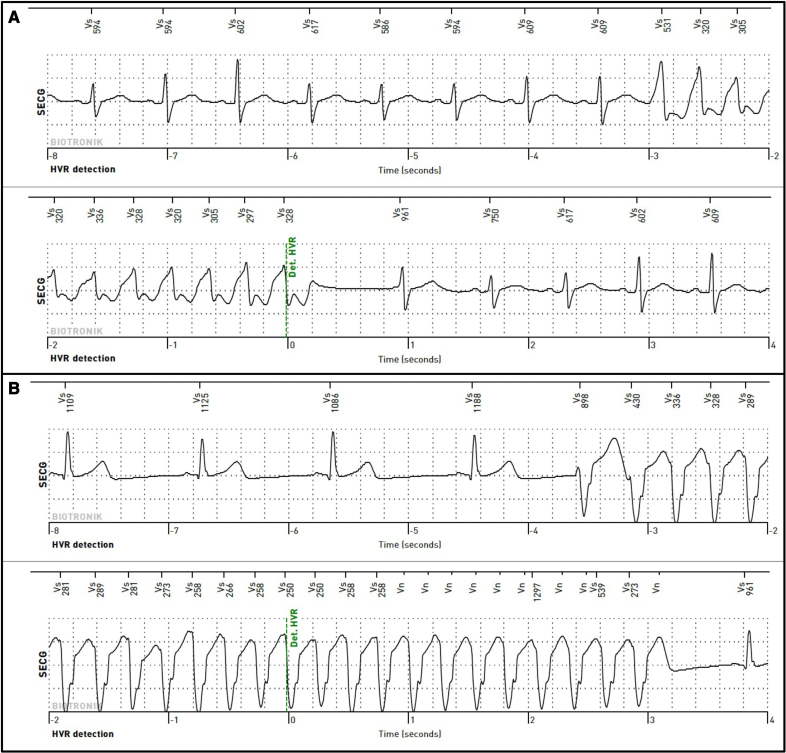
**Onset of ventricular arrhythmia on implantable loop recorder (ILR) monitoring.** ECG readings from athletes who developed ventricular arrhythmia during ILR monitoring. **A**, A 60-year-old cyclist who experienced nonsustained ventricular tachycardia (NSVT) at rest. **B**, A 64-year-old cyclist who developed asymptomatic NSVT at rest.

A total of 23/106 (21.7%) athletes experienced at least 1 ventricular arrhythmic episode with 3/106 (2.8%) having VT and 20/106 (18.9%) having nonsustained VT (Figure 3). All 3 participants who experienced VT were symptomatic and developed an episode of nonsustained VT before the onset of VT. They all underwent clinical evaluation by their local Consultant Cardiologist. Of those, 1 athlete received an implantable cardiac defibrillator due to presyncope, 1 was scheduled for an electrophysiology study and the third was advised to cease competing due to recurrent VT during exercise but declined further investigation (Table S3).

Of those who experienced a ventricular arrhythmia, 18/23 (78.3%) athletes had evidence of myocardial fibrosis on CMR compared with 5/23 (21.7%) athletes who did not (*P*<0.001). All athletes who experienced sustained VT had myocardial fibrosis on CMR. 12/106 (11.3%) athletes also experienced recurrent ventricular arrhythmia (≥2 episodes of nonsustained VT/VT) of whom 11/12 (91.7%) had myocardial fibrosis.

Athletes with ventricular arrhythmia on ILR monitoring also exhibited significantly greater LVEDVi (113±18 mL/m^2^ versus 106±13 mL/m^2^; *P*=0.04) and native T1 times (1252±46 ms versus 1241±39 ms; *P*=0.03) than those without ventricular arrhythmia. There were no significant differences in LV ejection fraction (55.8±4.2% versus 55.9±4.2%; *P*=0.92), RV size or right ventricular function between the groups. Nineteen of 23 (82.6%) athletes with ventricular arrhythmia had right ventricular insertion point (RVIP) LGE on CMR compared with 60/83 (72.3%) athletes with RVIP LGE who did not develop ventricular arrhythmia (*P*=0.32). There were no significant differences in T2 times between athletes with and without ventricular arrhythmia (41.0±2.2 ms versus 40.3±1.8 ms; *P*=0.21). No athlete had a T2 value >50 ms which would be indicative of acute myocardial edema, and no athlete fulfilled Lake Louise Criteria for acute myocarditis. Furthermore, there were no significant differences in any baseline characteristics or training history characteristics in athletes with and without ventricular arrhythmia (Table S4).

On 12-lead ECG, PVCs were more common in those athletes with ventricular arrhythmia than those without arrhythmia (3/23 [13.0%] versus 0/83 [0%]; *P*=0.001). However, the overall prevalence of ECG abnormalities was low. Athletes with ventricular arrhythmia also had a significantly higher prevalence of PVCs during exercise testing (19/23 [82.6%] versus 37/76 [48.7%]; *P*=0.004) with a greater burden of atypical PVC features (14/23 [60.9%] versus 18/76 [23.7%]; *P*<0.001).

### Predictors of Ventricular Arrhythmia

**Figure 4.**
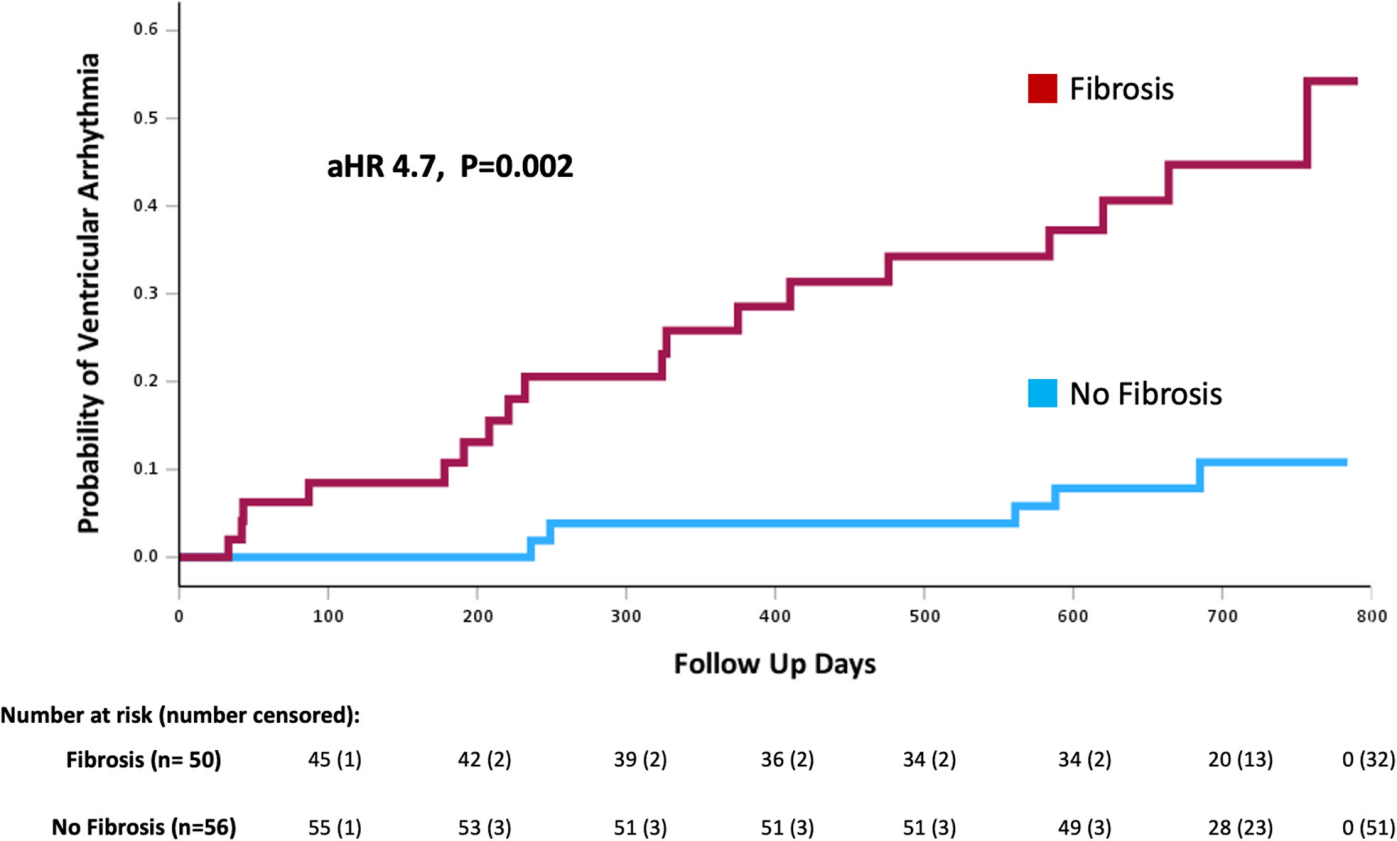
**Kaplan-Meier curve of probability of ventricular arrhythmia by myocardial fibrosis over time.** Kaplan-Meier curve demonstrating the increased risk of ventricular arrythmia in athletes with myocardial fibrosis (**top**, red) compared with those without myocardial fibrosis (**bottom**, blue line). aHR indicates adjusted hazard ratio.

By univariate Cox regression analysis, the presence of myocardial fibrosis was associated with an increased risk of ventricular arrhythmia (hazard ratio [HR], 4.7 [95% CI, 1.8–12.8]; *P*=0.002). Kaplan-Meier analysis also demonstrated athletes with myocardial fibrosis had a significantly higher probability of ventricular arrhythmia than those without fibrosis (log-rank test, *P*<0.001; Figure 4). The number of myocardial segments affected by fibrosis was associated with ventricular arrhythmia (HR, 1.66 [95% CI, 1.21–2.28]; *P*=0.002) along with the mass of fibrosis (HR, 1.14 [95% CI, 1.01–1.29]; *P*=0.04). Furthermore, LVEDVi (HR, 1.41 per 10 mL/m^2^ increase [95% CI, 1.05–1.88]; *P*=0.02), T2 (HR, 1.28 [95% CI, 1.02–1.62]; *P*=0.04), and PVCs during exercise testing (HR, 3.9 [95% CI, 1.32–11.4]; *P*=0.01) were associated with a greater risk of ventricular arrhythmia (Table 2).

**Table 2.**
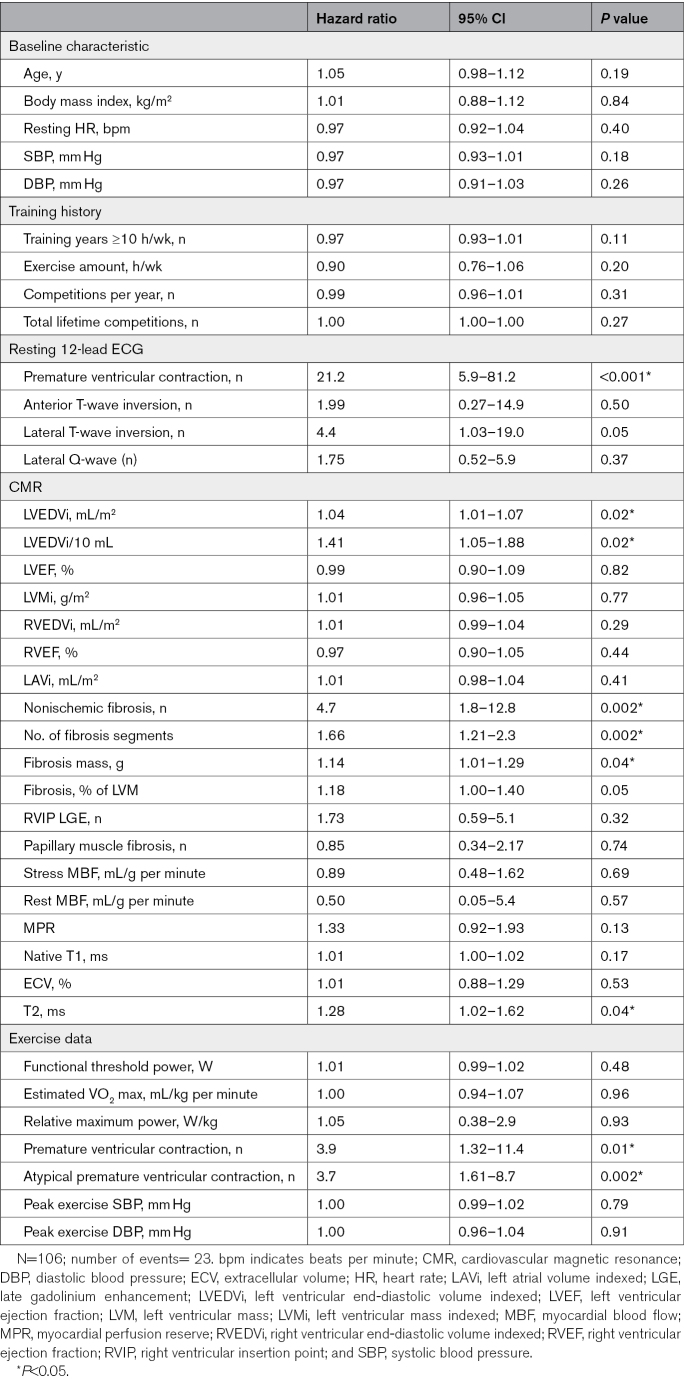
Univariate Cox Regression for Variables Associated With Incidence of Ventricular Arrhythmia

A multivariable Cox regression model including myocardial fibrosis and LV dilatation demonstrated both myocardial fibrosis (HR, 4.7 [95% CI, 1.7–12.7]; *P*=0.002) and LVEDVi (HR, 1.04 [95% CI, 1.01–1.07]; *P*=0.02) remained significantly associated with ventricular arrhythmia.

## Discussion

In this cohort of highly trained asymptomatic veteran male endurance athletes, nonischemic myocardial fibrosis was highly prevalent. Furthermore, the presence of nonischemic fibrosis (HR, 4.7), increased LVEDVi (HR, 1.41 per 10 mL/m^2^ increase), and PVCs during exercise (HR, 3.9) were associated with a greater risk of ventricular arrhythmia. The association between fibrosis and recurrent ventricular arrhythmia was even stronger, with fibrosis found in 11/12 of such athletes. Our findings cannot differentiate whether fibrosis itself is arrhythmogenic or a marker of an underlying cardiomyopathic process.

The prevalence of myocardial fibrosis in VENTOUX (VENTricular arrhythmia and cardiac fibrOsis in endUrance eXperienced athletes) was higher than previously reported including those studies which also excluded RVIP LGE from the definition of LV fibrosis.^10,11^ This may be explained by our study only including older male athletes (age was the variable most significantly associated with myocardial fibrosis). Furthermore, we studied cyclists and triathletes who undertook relatively high training volumes of endurance exercise over a long period. Therefore, the predefined inclusion criteria were designed to recruit a highly trained, older cohort who were most at risk of possessing myocardial fibrosis.

The presence of nonischemic myocardial fibrosis was over 4-fold greater in athletes than in a control group of age-matched nonathletes scanned with an identical CMR protocol. This suggests the high prevalence of fibrosis in athletes in our study is related to their sporting history rather than being a more general age-related finding.

As in previous reports, myocardial fibrosis in this study was predominantly found in the basal inferolateral LV segment.^4^ Prior myocarditis is regarded as a possible mechanism of basal inferolateral fibrosis in athletes due to similar CMR appearances.^12^ T2 values, which are typically raised in the acute phase of myocarditis, were not significantly raised in athletes with fibrosis, and no athlete fulfilled Lake Louise Criteria for acute myocarditis. Tahir et al^4^ found exercise-induced hypertension was independently associated with myocardial fibrosis in triathletes; however, this was not replicated in VENTricular arrhythmia and cardiac fibrOsis in endUrance eXperienced athletes.

We have reported a significant association between myocardial fibrosis on CMR and incident ventricular arrhythmia. It is unclear whether the presence of myocardial fibrosis on CMR is causally associated with ventricular arrhythmia or is a marker of another pro-arrhythmic pathological process. Athletes who developed ventricular arrhythmia had significantly greater LV cavity size than those who were free from ventricular arrhythmia. A 10 mL increase in LV cavity size was associated with a 40% increased risk of incident ventricular arrhythmia. Certain athletes may have a genetic predisposition to cavity dilatation, leading to a phenotypic overlap with dilated cardiomyopathy.^13^ In our study, myocardial fibrosis had a stronger association with the incidence of ventricular arrhythmia than LV dilatation. Furthermore, it is recognized that left-sided arrhythmogenic cardiomyopathy may manifest as lone LV fibrosis, as seen in athletes in this study.^14^ It is therefore possible that certain athletes with myocardial fibrosis and ventricular arrhythmia may have had concealed left-sided arrhythmogenic cardiomyopathy. However, all athletes in our study were asymptomatic at recruitment without known prior arrhythmia, were actively competing, and achieved above-average age-adjusted estimated VO_2_max on exercise testing. Furthermore, extracellular volume was lower in the athlete group compared with healthy nonathletic controls, thus suggestive of athletic remodeling rather than cardiomyopathy. While athletes with ventricular arrhythmia had significantly higher native T1 values than athletes without ventricular arrhythmia, T1 values in both athlete groups were well within the normal range and therefore unlikely to represent an underlying cardiomyopathy.

Our results also found RVIP LGE was widespread and not associated with ventricular arrhythmia. This supports the argument that RVIP is likely to be a benign finding in athletes which may be related to physiological enlargement of the right ventricular.

Ventricular arrhythmia is believed to be the main mechanism of SCD in athletes.^3^ Although the annual incidence of SCD during sport varies between studies, it is reported to be up to 6.8 per 100 000 athletes in certain cohorts.^15^ The annual rate of any ventricular arrhythmia in our study (10.9%) or sustained VT (1.4%) is considerably higher than SCD rates from the general population. Our study was not powered to detect SCD, which is substantially more rare, however asymptomatic ventricular arrhythmia is associated with increased mortality in the general population.^16^ Our reported incidence also reflects the strict study inclusion criteria, as we only recruited athletes with many years of cumulative exposure to intense training. Objective fitness assessment of VENTricular arrhythmia and cardiac fibrOsis in endUrance eXperienced athletes participants demonstrates they are among the fittest for their age. We also only recruited male athletes, who are over seven times more likely to suffer SCD than female athletes.^17^

### Clinical Implications of Myocardial Fibrosis in Athletes

CMR is increasingly used in the assessment of athletes, and our data suggest that myocardial fibrosis on LGE imaging is a relatively common finding in older male endurance athletes.^10^ Although the mechanisms are as yet unclear, our findings suggest that certain athletes with this finding are at increased risk of ventricular arrhythmia and should undergo risk stratification for future arrhythmic risk.

On univariate analysis both myocardial fibrosis (HR, 4.7) and PVCs during exercise (HR, 3.9) were associated with future ventricular arrhythmia, and we therefore propose that athletes with myocardial fibrosis identified on CMR should undergo exercise testing for further risk stratification.

Previous studies have utilized ECG and exercise testing as screening methods to identify symptomatic athletes to undergo CMR. Zorzi et al^8^ found athletes who presented with ventricular arrhythmia and were subsequently found to have nonischemic lateral LV wall fibrosis on CMR were more likely to experience malignant arrhythmic events than those without fibrosis. The same group investigated athletes presenting with exercise-induced ventricular arrhythmia and demonstrated this pattern of myocardial fibrosis was associated with a 7-fold increased risk of recurrent ventricular arrhythmia during exercise testing.^9^ VENTricular arrhythmia and cardiac fibrOsis in endUrance eXperienced athletes adds to this literature by prospectively demonstrating that myocardial fibrosis on CMR, LV dilatation, and PVCs during exercise are independent predictors of future ventricular arrhythmia.

### Limitations

The main limitation of our study is the limited sample size and highly selected nature of participants, and it remains to be established whether our findings in older, male, white European cyclists and triathletes would be replicated in other groups, including women and nonwhite athletes. Veteran male endurance athletes were specifically chosen as previous data identifies this group as those most likely to exhibit myocardial fibrosis on CMR and have the highest risk of SCD during sport. The high training requirement in the inclusion criteria led to recruitment of a highly selected group of athletes, and therefore, our findings do not support the use of CMR screening of unselected athletes. Athletes were recruited via self-referral using self-reported training history. While they denied symptoms and were screened for baseline CVD, it is possible that recruited athletes already harbored health concerns, thus leading to a referral bias. Furthermore, athletes did not undergo genetic testing, which may have identified concealed cardiomyopathy. The single-lead nature of the ILRs did not allow for the localization of ventricular arrhythmia to confirm that ventricular arrhythmia originated from the site of myocardial fibrosis. Finally, the end point of ventricular arrhythmia only indirectly correlates with SCD, and therefore, the clinical implications of our findings require further study.

### Conclusions

In male veteran endurance athletes, myocardial fibrosis was independently associated with the onset of ventricular arrhythmia. After adjusting for LV dilatation, which to a lesser extent was also associated with ventricular arrhythmia, fibrosis remained significantly associated with ventricular arrhythmia. Importantly, RVIP LGE was not associated with ventricular arrhythmia. Further studies are needed to establish whether fibrosis itself is arrhythmogenic or a marker of an underlying cardiomyopathic process.

## ARTICLE INFORMATION

### Acknowledgments

The authors thank David Broadbent (magnetic resonance imaging physicist), Jane Poh (medical student), and cardiovascular magnetic resonance radiographers at the Advanced Imaging Centre, in particular Lizette Cash and David Shelley, for their contributions.

### Sources of Funding

This research is supported by the National Institute for Health and Care Research (NIHR) Leeds Biomedical Research Center (NIHR203331), the British Heart Foundation, UK-PG/21/10322, and Leeds Clinical Research Facility.

### Disclosures

None.

### Supplemental Material

Supplemental Methods

Tables S1–S4

## Supplementary Material
