# Supplementary material for: Ventricular Arrhythmia and Cardiac Fibrosis in Endurance Experienced Athletes (VENTOUX)
Source: Circ Cardiovasc Imaging. 2025 Jul 17;18(8):e018470. doi: 10.1161/CIRCIMAGING.125.018470 (PMC12356567; doi:10.1161/CIRCIMAGING.125.018470)
Supplement: Supplementary file 1 [file hci-18-e018470-s001.pdf]

SUPPLEMENTAL MATERIAL

Table of Contents

1. Supplemental Methods 2

CMR Protocol 2

CMR Analysis 4

2. Supplemental Tables 6

Supplemental Methods

CMR Protocol

The scan comprised of the following stages detailed in chronological order:

1. Scout images to determine left ventricular (LV) short-axis (SAX); repetition time 3.4ms, echo time 1.4ms, flip angle 42°, matrix 208x140, 10-12 slices, 8mm slice thickness with 1.6mm gap, one cardiac phase.
2. LV function (cine) imaging in standard long-axis (LAX) (two-chamber, four-chamber and three-chamber views) and SAX planes to assess regional and global LV function using a breath-held balanced steady state free precession (SSFP) pulse sequence; repetition time 3.1-3.3m, echo time 1.4ms, flip angle 54°, matrix 208x140, one slice, 8mm slice thickness, 25 cardiac phases.
3. Pre-contrast native T1 maps acquired using a breath-held modified Look-Locker inversion recovery (MOLLI) acquisition; ECG triggered, 5s(3s)3s single-shot, SENSE factor 2, pre-pulse delay 180ms, repetition time 2.6ms, echo time 1.1ms, flip angle 20°, matrix 256x144, three SAX slices using the 3 of 5 slice selection method, slice thickness 8mm producing a reconstructed voxel size of 1.5x.5x8.0mm^3.
4. T2 maps acquired using a breath-held T2-prepared spoiled gradient echo (GE) pulse sequence resulting in single-shot T2 prepared images; T2 preparation duration 0,30 and 55ms, repetition time 3.6ms, echo time 1.3ms, flip angle 12°, matrix 192x116, three SAX slices using the 3 of 5 slice selection method, 8mm slice thickness producing a reconstructed voxel size of 2.0x2.0x8.0mm^3.
5. LV (cine imaging) volumes were acquired using a breath-held balanced SSFP pulse sequence covering the entire LV in the LV SAX; repetition time 3.1ms, echo time 1.4ms,

flip angle 52°, matrix 208x140, 15 slices, 8mm slice thickness with 2mm gap, 25 cardiac phases.

6. Adenosine stress perfusion imaging; 140 µg/kg/min of adenosine was administered through a peripheral intravenous cannula for a minimum of 3 minutes. Blood pressure (BP) was recorded every two minutes and continuous ECG monitoring was utilised throughout. When heart rate (HR) increased greater than 10% compared to baseline accompanied with symptoms of adenosine-induced hyperemia, a bolus of 0.05 mmol/kg non-ionic gadolinium-based contrast (Gadovist®) was given. Data was acquired with a dual sequence with separate pulse sequences for blood pool and myocardial tissue using a spoiled GE pulse sequence in three short axis slices using the three of five slice selection method (8mm slice thickness); 90 dynamic images per slice acquired; free-breathing with motion correction (MOCO) and pixel-wise mapping of myocardial blood flow (MBF). In-line automatic reconstruction and post-processing were implemented within the Gadgetron software framework.
7. Rest perfusion imaging after 6 minutes; pulse sequence and geometry was identical to stress imaging described above without administration of adenosine but a further bolus of 0.05 mmol/kg non-ionic gadolinium-based contrast (Gadovist®) was given.
8. Top up of further administration of 0.1 mmol/kg non-ionic gadolinium-based contrast (Gadovist®).
9. Look-Locker scout was performed to identify the optimal inversion time for LGE imaging using a T1-weighted, inversion-recovery balanced SSFP sequence; free-breathing with MOCO, repetition time 3.2ms, echo time 1.4ms, flip angle 47°, matrix 192x78, one slice, slice thickness 8mm.
10. LGE imaging using a T1-weighted, PSIR sequence in three standard LV LAX planes and SAX slices covering the entire LV 6 minutes after contrast; free-breathing with MOCO,

repetition time 2.6-2.7ms, echo time 1.1ms, flip angle 47°, matrix 256x144, SAX- 15 slices, slice thickness 8mm with 2mm gap producing a reconstructed voxel size of $1.6x1.6x8.0mm^3$.

11. Single slice of dark-blood LGE aiming to image both papillary muscles as per previously described LV imaging technique.
12. Post-contrast T1 mapping exactly 15 minutes after contrast administration using a 4s(3s)3s(3s)2s MOLLI acquisition with identical positioning and planning to the native T1 mapping.

CMR Analysis

Volumetric data were calculated by manually tracing the LV (endocardial and epicardial borders excluding papillary muscles, trabeculation and capillaries), right ventricle (RV) (endocardial borders) and left atrium (LA). LV/ RV volumes, LV mass and LA volumes were indexed to body surface area (BSA).

T1 and T2 maps were analysed by manually contouring the mid-LV slice endocardial and epicardial borders with a 15% offset applied to ensure blood pool was excluded. T1 measurements were performed on the same mid-LV slice using native T1 pre- and post-contrast images along with corresponding blood pool. ECV was calculated by using the following formula:

$$ECV = (1 - hematocrit)\,\frac{(\Delta R1 myocardium)}{(\Delta R1 blood)}$$

Conventional visual assessment of regional ischemia in a coronary distribution was performed from stress and rest perfusion images. Automated pixel-wise and segmental quantitative

measurement of stress and rest myocardial blood flow (MBF) and myocardial perfusion reserve (MPR) was performed.

The presence of focal LV LGE was confirmed only when an area of LGE was visualized on an LV SAX stack image along with a corresponding orthogonal LV LAX plane and matching ECV map image. LGE was interpreted as ischemic scar (myocardial infarction) when it involved the subendocardial layer or was transmural and followed a coronary distribution. Non-ischemic fibrosis was deemed present when LGE did not involve the subendocardial layer.

Fibrosis analysis was performed by two different level 3 certified CMR reporters who were blinded to the arrhythmia status of each participant. Segmental fibrosis analysis was performed using the 17-segment model and quantification was performed using the 5-standard deviation (SD) method to avoid overestimating the presence of subtle fibrosis. LV fibrosis percentage of total myocardium was calculated by dividing total LV fibrosis by LV mass. Isolated right ventricular insertion point (RVIP) LGE was noted but not classified as fibrosis. The presence of papillary muscle fibrosis was confirmed using a single slice of dark-blood LGE at the mid-LV level.

Exercise Test Starting Power

Exercise tests consisted of a 5-minute freestyle cycling warm-up followed by a maximal ramp-incremental test and ended with a 5-minute low intensity recovery ride. Starting power was determined by weight and competition level according to manufacturer's product guidance with incremental increases of 20 Watts (W) per minute until complete exhaustion was reached (Table 1).

Throughout the test, cadence, power output and HR were recorded to enable calculation of functional threshold power (FTP) output. Maximum HR estimated maximal oxygen consumption (VO_2max) and metabolic equivalent of tasks (METs) were also recorded. BP was recorded throughout the test to assess for abnormal BP response to exercise whilst ECG monitoring was also performed to identify arrhythmia and premature ventricular contractions (PVC). PVCs were characterised as atypical if they were multifocal, demonstrated R-on-T phenomena or occurred in couplets or greater. Data from external ECG and ILR were checked to ensure agreement.

Supplemental Tables

Table 1; Starting Power Determinants

Weight (kg)	Competition Level		
	Club (W)	National (W)	World Class (W)
<50	120	140	160
50-59	140	160	180
60-69	160	180	200
>69	180	200	220

Competition level was determined by the highest-ranking competition undertaken in the last year. kg, kilogram; W, Watts.

Table 2; CMR And Demographic Comparison Of Athletes And Age-Matched Controls

	**Athlete (n=106)**	**Control (n=27)**	**P value**
**Baseline Characteristic**			
Age (years)	59.2 ± 5.6	62.8 ± 7.3	0.09
Body Mass Index (kg/m^2)	24.9 ± 2.7	26.7 ± 2.3	**<0.001***
Resting Heart Rate (BPM)	53.3 ± 6.7	63.4 ± 9.1	**<0.001***
Systolic Blood Pressure (mmHg)	120 ± 11	130 ± 18	**<0.001***
Diastolic Blood Pressure (mmHg)	74.5 ± 7.2	78.4 ± 7.2	**0.01***
**CMR**			
LVEDVi (ml/m^2)	108 ± 15	82.1 ± 16.6	**<0.001***
LVEF (%)	55.7 ± 4.2	63.5 ± 4.3	**<0.001***
LVMi (g/m^2)	70.3 ± 9.6	57.0 ± 8.8	**<0.001***
RVEDVi (ml/m^2)	110 ± 17	88.1 ± 16.7	**<0.001***
RVEF (%)	52.9 ± 5.5	57.6 ± 7.2	**<0.001***
Non-Ischaemic Fibrosis (n)	50 (47.2%)	3 (11.1%)	**<0.001***
Ischaemic Fibrosis (n)	0 (0%)	1 (3.7%)	0.05
Basal Inferolateral LV Fibrosis (n)	44 (41.5%)	3 (11.1%)	**<0.003***
RVIP LGE (n)	79 (74.5%)	1 (3.7%)	**<0.001***
Stress MBF (ml/g/min)	2.2 ± 0.7	2.0 ± 0.5	0.12
Rest MBF (ml/g/min)	0.6 ± 0.2	0.7 ± 0.2	**0.04***
MPR	3.8 ± 1.2	3.1 ± 0.9	**0.01***
Native T1 (ms)	1242 ± 41	1271 ± 38	**<0.001***
ECV (%)	21.1 ± 2.1	23.6 ± 1.6	**<0.001***
T2 (ms)	40.4 ± 1.9	41.8 ± 2.8	**0.02***

Values are mean ± standard deviation or frequency (%). * P<0.05. Abbreviations: BPM, beats per minute; CMR; cardiac magnetic resonance; ECV, extracellular volume; LV, left ventricular; LVEDVi, left ventricular end-diastolic volume indexed; LVEF, left ventricular ejection fraction; LVM, left ventricular mass; LVMi, left ventricular mass indexed; MBF, myocardial blood flow; MPR, myocardial perfusion reserve; RVEDVi, right ventricular end-diastolic volume indexed; RVEF, right ventricular ejection fraction; RVIP, right ventricular insertion point.

Table 3; Clinical Details Of Ventricular Arrhythmic Events

Athlete	Age (y), Sport	NSVT episodes (n)	VT episodes (n)	Longest duration of NSVT/VT (beats)	Time to first incidence (days)	Symptoms	CMR findings	Adverse Outcome
1	64, cyclist	2	0	17	327	None	Normal LV/RV size & function Marked mid-wall basal inferior, inferolateral and anterolateral LGE.	No
2	58, cyclist	2	0	23	221	None	Dilated LV/RV size with borderline low function. Increased LV mass. Marked mid-wall and subepicardial basal inferolateral and anterolateral LGE.	No
3	56, cyclist	1	0	31	232	None	Severely dilated LV/RV size with normal function. Mid-wall basal inferolateral LGE.	No
4	66, cyclist	2	0	66	375	None	Normal LV/RV size & function. Marked mid-wall basal anterolateral LGE.	TIA
5	68, cyclist	1	0	3	191	None	Dilated LV with normal function. Normal RV size and function. Mid-wall basal inferolateral LGE.	No
6	64, cyclist	1	0	3	87	None	Normal LV/RV size & function. Subepicardial basal inferolateral LGE.	No
7	57, cyclist	1	0	28	757	None	Dilated RV with normal function. Normal LV size and function. Subtle mid-wall basal anterolateral LGE.	No
8	60, cyclist	2	0	10	208	Pericarditic chest pain at rest but non-raised Troponin T	Severely dilated LV/RV size with borderline low function. Increased LV mass. Marked mid-wall and subepicardial basal inferior and inferolateral LGE.	No
9	53, triathlete	1	0	17	588	None	Normal LV/RV size & function. No LGE.	No

10	59, cyclist	1	0	27	620	None	Dilated LV/RV size with normal function. Marked mid-wall and subepicardial basal inferolateral and anterolateral LGE.	No
11	69, cyclist	3	0	16	324	None	Dilated LV/RV size with normal function. Mid-wall and subepicardial basal inferolateral LGE.	No
12	61, cyclist	4	0	27	410	None	Normal LV/RV size & function Mid-wall basal inferior, inferolateral and anterolateral LGE.	No
13	58, cyclist	1	0	10	236	None	Dilated LV/RV size with normal function. No LGE.	No
14	71, cyclist	2	1	3 minutes	43	Palpitations and pre-syncope causing to stop exercise	Dilated RV with impaired function. Normal size LV and function. Marked mid-wall basal inferolateral LGE.	Listed for ICD on basis of VT length and symptoms
15	52, triathlete	1	0	17	584	None	Normal LV size & function. Normal RV size but borderline low function. Mid-wall basal inferolateral and anterolateral LGE.	No
16	63, cyclist	7	1	44 seconds	42	Reduced exercise capacity and dyspnoea during race	Severely dilated LV/RV size with borderline low function. Increased LV mass. Marked subepicardial basal inferolateral and anterolateral LGE.	Advised to cease competing- Declined further tests
17	53, triathlete	1	0	3	685	None	Borderline dilated LV/RV size with borderline low function. No LGE.	No
18	62, triathlete	1	0	3	664	None	Dilated LV/RV size with normal function. Increased LV mass. Mid-wall basal inferolateral and anterolateral LGE.	No

19	55, cyclist	2	0	28	249	None	Dilated LV/RV size with borderline low LV function and normal RV function. No LGE.	No
20	52, cyclist	1	0	34	561	None	Borderline dilated LV size with borderline low function. Dilated RV with impaired function. No LGE.	No
21	64, cyclist	3	0	12	178	None	Dilated LV/RV size with normal function. Subtle mid-wall basal inferolateral LGE.	No
22	64, cyclist	2	0	6	476	None	Severely dilated LV/RV size with borderline low LV function and normal RV function. Increased LV mass. Mid-wall and subepicardial basal and inferolateral LGE.	No
23	51, triathlete	2	1	8 minutes	33	Palpitations and dyspnoea after exercise	Dilated LV/RV size with borderline low LV function and normal RV function. Subtle mid-wall basal anterolateral LGE.	Awaiting EP study

% LGE expressed as LGE mass/ LV mass. Abbreviations: CMR, cardiac magnetic resonance; EP, electrophysiology; ICD, implantable cardiac defibrillator; LGE, late gadolinium enhancement; LV, left ventricular; NSVT, non-sustained ventricular tachycardia; RV, right ventricular; VT, ventricular tachycardia

Table 4; Variables According To The Incidence Of Ventricular Arrhythmia

	**No VA (n=83)**	**VA (n=23)**	**P value**
**Baseline Characteristic**			
Age (Years)	58.9 ± 5.6	60.4 ± 5.8	0.22
Body Mass Index (kg/m^2)	24.8 ± 2.8	25.1 ± 2.7	0.61
Resting Heart Rate (BPM)	54.5 ± 7.1	52.5 ± 7.1	0.80
Systolic Blood Pressure (mmHg)	121 ± 11	118 ± 11	0.21
Diastolic Blood Pressure (mmHg)	74.8 ± 71	73.0 ± 7.5	0.24
**Training History**			
Training Years (≥ 10 Hours Per Week)	20.0 (12.9 – 30.0)	15.0 (10.0 – 20.0)	0.08
Weekly Training (Hours)	12.0 ± 3.4	11.0 ± 2.0	0.31
Competitions Per Year	20.0 (4.0 – 30.0)	20.0 (12.0 – 25.0)	0.48
Total Lifetime Competitions	360 (147 – 600)	200 (168 – 495)	0.15
**Resting 12-Lead ECG**			
Premature Ventricular Contraction (n)	0	3 (13.0%)	**0.009***
Anterior T-wave Inversion (n)	1 (1.2%)	1 (4.4%)	0.39
Lateral T-wave Inversion (n)	1 (1.3%)	2 (8.7%)	0.12
Anterior Q-wave (n)	2 (2.4%)	0	1.00
Lateral Q-wave (n)	5 (6.0%)	3 (13.0%)	0.37
**CMR**			
LVEDVi (ml/m^2)	106 ± 13	113 ± 18	**0.04***
LVEF (%)	55.9 ± 4.2	55.8 ± 4.2	0.92
LVMi (g/m^2)	70.0 ± 9.6	71.2 ± 9.9	0.61
RVEDVi (ml/m^2)	109 ± 17	113 ± 18	0.35
RVEF (%)	53.1 ± 5.2	52.2 ± 6.7	0.46
LAVi (ml/m^2)	44.8 ± 13.4	46.9 ± 11.5	0.39
Non-Ischemic Fibrosis (n)	32 (38.6%)	18 (78.3%)	**<0.001***
Number Of Fibrosis Segments	1.7 ± 0.9	1.8 ± 0.9	0.45
Fibrosis Mass (g)	2.9 ± 2.6	2.8 ± 1.8	0.67
% Fibrosis Of Total LVM (%)	2.1 ± 1.8	1.9 ± 1.2	0.97
Basal Inferolateral LV Fibrosis (n)	28 (33.7%)	16 (69.6%)	0.89

RVIP LGE (n)	60 (72.3%)	19 (82.6%)	0.32
Papillary Muscle Fibrosis (n)	24 (28.9%)	6 (26.1%)	0.79
Segmental Inducible Ischemia (n)	0	0	NA
Stress MBF (ml/g/min)	2.2 ± 0.7	2.1 ± 0.8	0.75
Rest MBF (ml/g/min)	0.6 ± 0.2	0.6 ± 0.2	0.24
MPR	3.7 ± 1.1	4.0 ± 1.5	0.27
Native T1 (ms)	1241 ± 39	1252 ± 46	**0.03***
ECV (%)	21.0 ± 2.0	21.2 ± 2.1	0.76
T2 (ms)	40.3 ± 1.8	41.0 ± 2.2	0..21
**Exercise Data**			
Maximum Power (W)	382 ± 51	386 ± 57	0.71
Relative Maximum Power (W/kg)	4.9 ± 0.7	4.9 ± 0.9	0.66
Functional Threshold Power (W)	241 ± 29	248 ± 29	0.32
METs (kcal/kg/hour)	16.3 ± 1.7	16.6 ± 1.8	0.47
Estimated VO_2 max (mL/kg/min)	51.3 ± 6.7	51.3 ± 6·8	0.97
Premature Ventricular Contraction (n)	37 (48.7%)	19 (82.6%)	**0.004***
Atypical Premature Ventricular Contraction (n)	18 (23.7%)	14 (60.9%)	**<0.001***
Peak Exercise Maximum Heart Rate (BPM)	168 ± 12	162 ± 13	**0.03***
Peak Exercise Systolic Blood Pressure (mmHg)	197 ± 26	199 ± 22	0.79
Peak Exercise Diastolic Blood Pressure (mmHg)	89.6 ± 10.9	89.6 ± 9.1	0.63

Values are mean ± standard deviation or frequency (%). * P<0.05. Abbreviations: BPM, beats per minute; CMR; cardiac magnetic resonance; ECV, extracellular volume; LAVi, left atrial volume indexed; LVEDVi, left ventricular end-diastolic volume indexed; LVEF, left ventricular ejection fraction; LVM, left ventricular mass; LVMi, left ventricular mass indexed; MET, metabolic equivalent of task; MBF, myocardial blood flow; RVEDVi, right ventricular end-diastolic volume indexed; RVEF, right ventricular ejection fraction; RVIP, right ventricular insertion point; VA, ventricular arrhythmia. (N=99 for PVC and atypical PVC during exercise test- No VA=76 and VA=23).
